# Good practice guidelines for biomarker discovery from array data: a case study for breast cancer prognosis

**DOI:** 10.1186/1752-0509-7-S4-S2

**Published:** 2013-10-23

**Authors:** Jie Cheng, Joel Greshock, Leming Shi, Shu Zheng, Alan Menius, Kwan Lee

**Affiliations:** 1 Quantitative Sciences, GlaxoSmithKline, Collegeville, PA 19426, USA; 2 Cancer Research, GlaxoSmithKline, Collegeville, PA 19426, USA; 3 National Center for Toxicological Research, US Food and Drug Administration, Jefferson, AR 72079, USA; 4 Cancer Institute, Zhejiang University, Hangzhou, 310009, China

## Abstract

**Background:**

Biomarker discovery holds the promise for advancing personalized medicine as the biomarkers can help match patients to optimal treatment to improve patient outcomes. However, serious concerns have been raised because very few molecular biomarkers or signatures discovered from high dimensional array data can be successfully validated and applied to clinical use. We propose good practice guidelines as well as a novel tool for biomarker discovery and use breast cancer prognosis as a case study to illustrate the proposed approach.

**Results:**

We applied the proposed approach to a publicly available breast cancer prognosis dataset and identified small numbers of predictive markers for patient subpopulations stratified by clinical variables. Results from an independent cross-platform validation set show that our model compares favorably to other gene signature and clinical variable based prognostic tools. About half of the discovered candidate markers can individually achieve very good performance, which further demonstrate the high quality of feature selection. These candidate markers perform extremely well for young patient with estrogen receptor-positive, lymph node-negative early stage breast cancers, suggesting a distinct subset of these patients identified by these markers is actually at high risk of recurrence and may benefit from more aggressive treatment than cur-rent practice.

**Conclusion:**

The results show that by following good practice guidelines, we can identify highly predictive genes in high dimensional breast cancer array data. These predictive genes have been successfully validated using an independent cross-platform dataset.

## Introduction

The goal of biomarker discovery from high dimensional array data is to find an individual or a set of genes (or any other molecular variables) whose expression pattern can predict certain phenotype or clinical outcome. Biomarker discovery holds the promise for advancing personalized medicine as the biomarkers can help match patients to optimal treatment and thus improve patient outcomes. During the past 15 years, numerous biomarkers and gene signatures have been published in the literature. However, few of these biomarkers can be successfully validated and applied in clinical setting, which have caused serious concerns in biomedical research community [1].

The lack of success in biomarker discovery is mainly due to three issues.

(1) Many published gene signatures cannot be validated independently. This is mainly due to flawed data analysis. For example, fail to keep completely untouched validation data or improperly use cross-validation technique can all cause overly optimistic results being reported.

(2) Some gene signatures do not have additional clinical benefit over known clinical variables even though the gene signatures can be validated. For example, gene signatures discovered from a breast cancer patient population including both ER + and ER- patients for predicting treatment response could probably end up approximating ER status, an important clinical variable for predicting treatment response.

(3) Some gene signatures contain large numbers of genes making them difficult to be applied in clinical setting. These gene signatures may include lot of unimportant genes due to the inefficiency of the standard feature selection techniques. For example, standard t test will either miss or rank certain important features below many non predictive features, if these important features do not conform to normality.

The first two issues have been addressed in [1,2]. To address the third issue, we developed a two-way filtering based method to parsimoniously identify the most informative features from different types of distributions. Our method selects features by searching for the desired thresholds of a pair of statistics that are used to filter features. For any pair of thresholds, the features that satisfy both thresholds are used to build a diagonal linear discriminate analysis (*DLDA*) classifier [3]. When choosing the pair of statistics, we choose one that is more efficient at detecting strong signals such as mean difference test, and a second that is more efficient at controlling signal to noise ratio such as classical t-test or Mann-Whitney U test. By varying the thresholds of these two statistics in certain steps within their acceptable ranges, we can achieve various tradeoffs and control the size of the feature sets. The method and other supporting functions are implemented in a Java based tool called *Array Data Analyzer (ADA*). The detailed method is described in [4].

Compared to standard feature selection tools, our *ADA *tool has the following advantages.

(1) Because our approach does not select features based on any single statistic or fixed tradeoff of two statistics, it can adapt to different data and parsimoniously identify informative features from different distributions. Experimental result presented in [4] shows that our approach yields much smaller models on average when compared to other standard approaches, yet achieving similar or better performance.

(2) By using grid-search to explore different tradeoffs of a pair of statistics, our tool is very flexible to address different feature selection requirements in real world situations. For example, researchers at times are willing to sacrifice performance in order to gain other logistic properties, such as smaller numbers of features and larger fold change. These properties enable more tractable assays for clinical use (e.g. qPCR). By visually examining the performance at different combinations of cut points of the two statistics and checking the size of feature sets, researchers can decide which feature set would be best to use.

(3) Our Java based tool is very efficient to run even though it conducts rigorous cross validation and grid search to find optimal feature sets. For example, finding an optimal gene signature from microarray gene expression dataset with 130 samples takes about 50 seconds on a modest laptop PC (Dell Latitude E4300 with 2GB of RAM). (Searching through 200 combinations of the threshold pairs of the two statistics; using 5 times 5 fold cross validation (CV) to measure performance of each combination).

The *ADA *tool has been applied to various GSK drug discovery projects [5]. It has also been applied to the FDA MAQC-II project [6] by the GSK data analysis team (DAT). The GSK DAT achieved highest mean area under the receiver operating characteristic curve (*AUROCC*) across all 11 endpoints among the participating DATs. Using *ADA *tool, we also achieved the second place in the "The Sage Bionetworks/DREAM breast cancer prognosis challenge 2012" (https://sagebionetworks.jira.com/wiki/display/BCC/Home) by simply identifying 8 genes from the provided training set.

As a case study, we performed detailed prognostic analyses on the van de Vijver breast cancer dataset [7]. An independent cross-platform validation set TRANSBIG [8] was then used to validate our findings. The results show that our 20-gene signature as well as many of the discovery individual prognostic biomarkers can achieve comparable or better performance compared to the clinical or gene signature based prognostic scores. These discovered biomarkers have the potential to be used in clinical settings to identify a subset of the lymph-node-negative (Node-) and estrogen-receptor-positive (ER+) patients who are at a higher risk of relapse.

## Methods

### The *ADA *tool

The *ADA *tool allows users to choose different pairs of statistics to perform the two-way filtering. In this paper, the default pair of non parametric statistics is used, which are the mean difference test and the Mann-Whitney U test. More detailed information of the ADA tool is given in [4].

### Data preprocessing

van de Vijver dataset and TRANSBIG dataset were downloaded from the public domain. For all datasets generated from Affymetrix platforms, if a gene intensity value is smaller than 40, we floor it to 40. The log transformed ex-pression values are used for analysis.

### Scoring and classification of validation data sets using 20 gene signature

For the discovered genes that can be successfully mapped from Agilent Hu25K platform to Affymetrix HG-U133A, we arbitrarily decided to keep top 10 genes from each direction (i.e., over expression → poor prognosis and over expression → good prognosis) to validate. If a gene can be mapped to multiple probe sets of the Affymetrix HG-U133A chip, we use the probe set that has the largest interquartile range (IQR). To avoid bias, the scoring of validation samples using the 20 gene signature was done without any scaling or tuning. We simply calculated the scores by summing up the log expression values of genes of one direction and subtracting those of genes of the other direction. To classify the samples into "good prognosis" and "poor prognosis", we use zero as the threshold.

## Results

As a case study, we use the van de Vijver data set [7] to discover prognostic biomarkers for various patient subsets stratified by clinical variables. The markers discovered from the lymph node negative patient cohort are subsequently evaluated using an independent cross-platform dataset: TRANSBIG [8]. Table 1 summarizes the training and independent validation data sets. Both data sets are publicly available.

**Table 1:** Summary of the two data sets involved in this experiment

Data set	van de Vijver	TRANSBIG
**Purpose in this experiment**	biomarker discovery	biomarker validation
**Microarray platform**	Agilent Hu25K	Affymetrix HG-U133A
**No. of Patients**	295	198
**Age**	<53	<61; mean = 46
**ER status (pos/neg)**	226/69	134/64
**Lymph node (pos/neg)**	144/151	0/198
**Systemic treatment**	some	none
**Data location or GEO accession**	http://bioinformatics.nki.nl/data.php	GSE7390

### Model training and prognostic biomarker discovery using van de Vijver dataset

The van de Vijver data set contains samples from 295 patients with stage I and II breast cancer. The gene expression data was generated using Agilent Hu25K platform. A subset of the samples was used to develop a 70-gene signature for predicting breast cancer early relapse [9]. To make the data less noisy, we only included patients who developed metastases in < 5 years and those who remained disease free for > 10 years (i.e. we removed the patients who developed metastases between 5-10 years) in the biomarker discovery phase. This left 78 patients who developed metastases within 5 years (poor prognosis) and 68 patients who remain disease free for at least 10 years. Besides analyzing all the data together, we also utilized the clinical information (ER status and lymph node status) to create subgroups of samples in order to search for prognostic markers within these clinical subgroups.

By using the *ADA *tool, we were able to generate gene lists of various sizes for different subgroups of patients. After studying the gene lists, we found that many of the predictive markers were indeed more sig-nificant in certain subgroups. For example, the ER+/lymph node+ group harbors unique genes that are predictive of relapse. The prognosis of this group of patients was also more predictable (based on nested CV results) compared to that of the other groups. Some of the discovered genes are listed in Table 2.

**Table 2:** Candidate markers identified from the van de Vijver data set using the proposed method

Group	Sample size n (good prog + poor prog)	Nested CV *AUROCC*performance	**Feature list (high expression **→ **poor prognosis)**	**Feature list (high expression **→ **good prognosis)**
**All patient**	**146 (68+78)**	**0.73 (0.04)**	BIRC5, CCNB2, CENPA, TK1, CCNE2, DKFZp762E1312, PRC1, STK15, SLC16A3, BUB1	CEGP1, SLC11A3, C4A, ZNF145, MATN3, PGR, RAI2, DLX2

**ER+**	**107 (57+50)**	**0.76 (0.05)**	H1F2, COX6C, H2BFB, CCNE2, BLVRB	FST, DIO3, NTN4, DLX2, MATN3, COL3A1

**Node+**	**64 (30+34)**	**0.80 (0.06)**	H1F2, H2BFB, HA2FO, H2AFA, HABFB, KFZp762E1312, H2BFS	LTF, NTN4, HML2, PER1, DMBT1, ODZ2, WNT5A, SEMA3C

**Node-**	**82 (38+44)**	**0.72 (0.06)**	PRAME, FADSD6, TK1, TSSC3, CTSL2, BUB1	CEGP1, ESR1, CYP4B1, SEC14L2, TBX3-iso, ZNF145

**ER+/Node+**	**50 (26+24)**	**0.83 (0.06)**	H1F2, H2BFB, H2AFP, H2AFA, H2BFB, COX6C, MSMB, BLVRB, , BCAS1	LTF, LAMB3, C4A, NTN4, PTPRK, RTN1

Many genes discovered in larger groups can also be discovered in their subgroups. For example, BIRC5 can be discovered in most of the subgroups. These genes are not listed again in subgroups unless they are more significant in the subgroups. A gene may be listed in a larger group only because it is significant in one of its subgroups. For example, H1F2 is listed in lymph node-positive group only because it is significant in ER+/Node+ subgroup. The nested CV performance is listed with estimated standard error.

### Independent cross platform validation using TRANSBIG dataset

To validate the candidate biomarkers discovered from van de Vijver data set, we download the TRANSBIG data set (198 patients with mean age equals 46), which is based on the Affymetrix HG-U133A platform instead of the Agilent Hu25K platform. An original objective of TRANSBIG data set was to validate the 70-gene signature and it is similar to the van de Vijver data set in term of patient age distribution - i.e., both data sets contains young breast cancer patients. Because all patients of the TRANSBIG data set are lymph node negative (Node-) patients, we evaluate the candi-date biomarkers discovered from the Node- patients.

We picked the top 20 genes from our Node- gene list that can be mapped from Agilent Hu25K to Af-fymetrix HG-U133A. The performance of the 20 gene-signature as well as each of the individual gene was measured using *AUROCC *based on commonly used endpoints: time to distant metastasis(TDM) at 5 years and 10 years. Because 70% of Node- patients in the training data belong to the subgroup Node-/ER+, in addition to measure the performance on the whole validation sets (consisting of Node- patients), we also measured the performance on the Node-/ER+ subset of patients. Both results are shown in Table 3, which also includes the performance of two commonly used clinical scores (Nottingham Prognostic Index Score [10] and Adjuvant! 10 year overall survival score [11]), three clinical variables (tumor grade, tumor size and age), two known markers (ESR1 and MKI67) and three gene signatures (16-gene signature [12], 70-gene signature [9] and 76-gene signature [13]).

**Table 3:** Validation performance (*AUROCC*) of the candidate biomarkers in TRANSBIG data sets

Prognostic factors	TRANSBIG			
	
	TDM at 5yrs		TDM at 10 yrs	
	Node-	Node-/ER+	Node-	Node-/ER+
202705_at(CCNB2)	**0.74**	**0.83**	**0.72**	**0.80**
209642_at(BUB1)	**0.71**	**0.81**	**0.70**	**0.78**
204962_s_at(CENPA)	**0.69**	**0.84**	**0.69**	**0.79**
203362_s_at(MAD2L1)	**0.68**	**0.75**	**0.67**	**0.71**
202095_s_at(BIRC5)	**0.67**	**0.78**	**0.65**	**0.74**
210074_at(CTSL2)	**0.65**	**0.64**	**0.65**	**0.64**
209803_s_at(PHLDA2, TSSC3)	**0.61**	0.62	0.59	0.61
202338_at (TK1)	**0.61**	**0.69**	0.60	**0.64**
204086_at(PRAME)	**0.61**	0.62	0.57	0.58
202218_s_at (FADSD6)	0.50	0.49	0.50	0.45

*210096_at(CYP4B1)*	**0.71**	**0.78**	**0.70**	**0.74**
*205883_at(ZNF145)*	**0.69**	**0.75**	**0.66**	**0.71**
*219197_s_at(SCUBE2, CEGP1)*	**0.66**	**0.66**	**0.63**	0.59
*214053_at(ERBB4)*	**0.66**	**0.72**	**0.67**	**0.74**
*208305_at(PGR)*	**0.65**	**0.66**	**0.64**	**0.66**
*219682_s_at(TBX3)*	**0.63**	**0.66**	**0.63**	**0.65**
*204541_at(SEC14L2)*	**0.63**	**0.65**	**0.62**	0.59
*206091_at(MATN3)*	**0.61**	0.55	**0.61**	0.56
*202554_s_at(GSTM3)*	**0.60**	0.62	0.58	0.59
*219440_at(RAI2)*	0.59	0.59	0.56	0.56

**Our 20-gene signature**	**0.73**	**0.83**	**0.70**	**0.79**
16-gene signature	**0.71**	**0.79**	**0.69**	**0.73**
70-gene signature	**0.68**	NA	NA	NA
Nottingham Prognostic Index Score	**0.67**	**0.68**	**0.66**	**0.66**
Adjuvant! Online 10 year OS prob.	**0.66**	**0.64**	**0.67**	**0.63**
76-gene signature	**0.65**	**0.68**	**0.62**	**0.64**
Tumor grade	**0.64**	0.63	**0.62**	0.62
Tumor Size	**0.63**	**0.65**	**0.63**	**0.64**
212021_s_at(MKI67)	**0.62**	**0.70**	**0.65**	**0.70**
205225_at (ESR1)	0.58	0.59	0.57	0.61
Age	0.53	0.47	0.52	0.51

There are two *AUROCC *numbers for each gene at each endpoint. The first number is from the whole validation set with 100% Node- patients; the second is from the Node-/ER+ subset of the validation set. The numbers in bold font are significant at 95% confidence level. The top portion of the table contains 10 genes of one direction (over expression → poor prognosis). The middle portion contains 10 genes of the opposite direction (over expression → good prognosis). The bottom portion contains our signature based on all 20 genes and other prognostic factors. The performance of the 70-gene signature for the TRANSBIG data set is copied from [8]. The performance of the 76-gene signature is based on binary prediction of "good prognosis" and "poor prognosis" for each patient. Among the listed 20 genes, three genes (CENPA, GSTM3 and CEGP1) were included in the 70-gene signatures [9] and four genes (BIRC5, PGR, SCUBE2 and CTSL2) were included in the 16-gene signature. There is no overlap between our 20-gene signature and the 76-gene signa-ture.

From the result we can see that our 20-gene signature performs better than other gene signature or clinical variable based prognostic factors. Most of the 20 candidate genes can also be successfully validated. About half of the 20 genes can individually achieve similar or better performance compared to clinical variable based risk scores, which further shows that the proposed approach can parsimoniously select high quality features.

**Figure 1:**
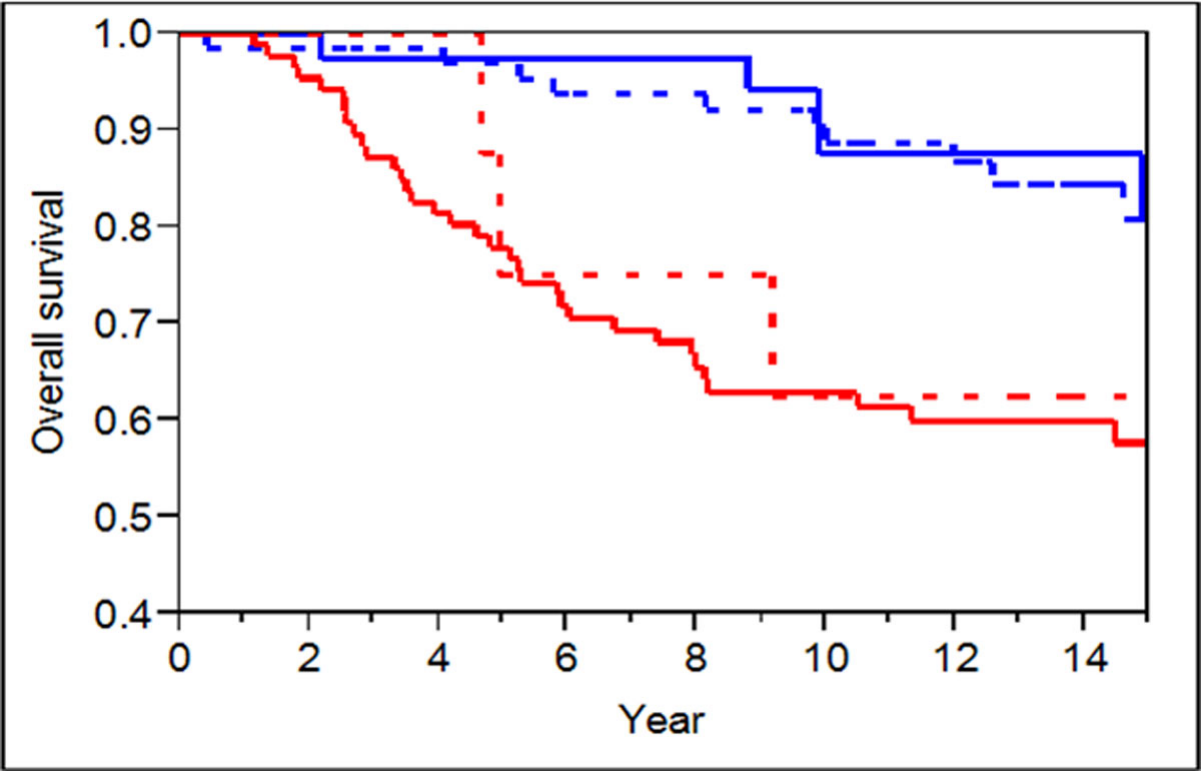
**Using Kaplan-Meier curves to compare 20-gene signature with the Adjvant! 10 year overall sur-vival score**. Solid blue curve is for the risk group that both 20-gene signature and Adjvant! predict good prognosis (n = 38); Dotted blue curve is for the risk group that 20-gene signature predicts good prognosis and Adjvant! predicts poor prognosis(n = 66); Dotted red curve is for the risk group that 20-gene signature predicts poor prognosis and Adjvant! predicts good prognosis(n = 8); Solid red curve is for the risk group that both classifiers predict poor prognosis (n = 86).

To further illustrate the added value of these markers over the traditional clinical criteria, we use survival curves to compare the 20-gene signature to the Adjvant! 10 year overall survival probability, which is based on known clinical markers. Figure 1 plots four Kaplan-Meier curves by dividing the TRANSBIG patients into four risk groups based on the predictions from 20-gene signature and the Adjvant! tool. The plots show that our 20-gene signature is superior in predicting cancer outcome, especially for the 66 patients (33% of the 198 samples) shown in the dotted blue survival curve where the 20-gene signature predicts good outcome and Adjvant! predicts poor outcome. This curve indeed traces the curve in solid blue where the patients were predicted to have good outcome by both classifiers.

It is also worth noting that the 20-gene signature and the top performing genes based on the whole vali-dation set have even better performance when validated using Node-/ER+ patients only. For example, for end point TDM at 5 years, the 20-gene signature can achieve *AUROCC *0.83; and the genes CCNB2, BUB1, CENPA, BIRC5 and CYP4B1 can each achieve *AUROCC *above 0.78.

## Discussion

Using the breast cancer prognosis experiment, the *ADA *tool was able to effectively discover both biologically relevant and class predictive genes. The tool has two important features: (a) because the model performance estimation is based on rigorous nested cross-validation, the tool does not return any feature when the dataset does not produce a strong signal. (b) It has a unique iterative procedure that attempts to find all important genes, which can be used for further analyses, such as pathway and ontology analysis.

When doing biomarker discovery using the van de Vijver datasets, our approach relies heavily on robust cross validation to control over fitting rather than leaving out an artificial validation set. This is a more efficient way to use the relatively small number of samples - artificially defining a small validation set is unlikely to achieve much due to the large test data variability and reduced power of model development in the smaller training set. However, it is crucial that the cross-validation is done properly. For example, the feature selection must be performed within each run of cross validation; and the nested cross validation is often required when evaluating model performance (i.e., outer loop CV for model performance evaluation and inner loop CV for model parameter tuning). As we can see here, the proper cross-validation procedures can often be quite computationally expensive. This is one of the reasons that a simple modeling technique such as *DLDA *is preferred. We believe the best way to gain better performance is through improving performance of feature selection, rather than tuning modeling parameters of complex models. Complicated learning schemes can make proper cross-validation too computational expensive to run. Without proper cross-validation, the result can be overly optimistic. Important guidelines for biomarker discovery are presented in Richard Simon's work [2].

As shown in Table 2, when sample size allows, we try to discover biomarkers for patient subpopulations stratified by clinical variables. This also assures us that the discovered biomarkers indeed have added value over the traditional clinical variables, rather than approximating the clinical variables.

To validate our findings from the van de Vijver data, we used the cross-platform validation dataset TRANSBIG. In addition to evaluate our 20-gene signature, we also evaluate individual genes. By doing so, we can carry forward those validated genes for further clinical validations. Table 3 shows that most of the 20 genes can be validated using the TRANSBIG data set and some of the discovered candidate genes can individually achieve similar or better performance compared to the state-of-the-art breast cancer prognostic tests, which suggest that our 20-gene signature contains mostly highly predictive genes. It is very likely that large gene signatures contain many unimportant genes. We believe that a promising way to improve prediction accuracy is to combine gene signatures with traditional clinical variables. This can be achieved by developing gene signatures containing a small number of genes for each clinically homogeneous subset of patients.

Table 3 also shows that for Node-/ER+ patients of younger age (as those in TRANSBIG dataset), there are a number of genes that can predict cancer recurrence at high accuracy. This suggests that there is a distinct subgroup of Node-/ER+ patients who have high risk of relapse and should be treated more aggressively. Our 20-gene signature is acquired from analyzing one public dataset for one patient subpopulation (Node-). We plan to analyze other datasets to refine this predictive gene list and develop gene lists for other patient subpopulations.

Based on our past experience, we are confident that biomarker discovery can play important role in ad-vancing personalized medicine if the study design and data analysis is done properly. We would like to propose the following good practice guidelines for biomarker discovery. Most of the points have already been made in [1,2].

(1) Make sure no information leak from validation set. If a separate validation set is available, one need to make sure that the validation set is not used in any way in feature filtering or model building. The same rule also applies to cross validation.

(2) Model performance evaluation and model parameter tuning cannot be done using the same cross va-lidation loop. In such case, nested cross validation is needed (i.e., outer loop CV for model performance evaluation and inner loop CV for model parameter tuning).

(3) When sample size is not very large, cross validation is a preferred validation technique.

(4) Consider patient stratification using known clinical variables.

(5) Use simple modeling techniques. Simple models performs well [3] and are easy to train and easy to un-derstand.

(6) When choosing feature filtering or feature selection methods, do not automatically assume that all va-riables are normally distributed. Many highly predictive biomarkers are far from normally distributed, especially in cancer research [14,15].

## Abbreviations

*AUROCC*: area under the receiver operating characteristics curve; CV: cross-validation; DAT: data analysis team; *DLDA*: diagonal linear discriminant analysis; ER: estrogen receptor; MAQC-II: MicroArray Quality Control phase II on predictive modeling.

## Competing interests

The authors declare that they have no competing interests.

## Authors' contributions

JC designed and implemented the proposed feature selection algorithm and performed the experiments. All authors participated in discussing and drafting the manuscripts. All authors participated to and approved the final manuscript's preparation.

## Declarations

The publication costs for this article were funded by the corresponding author’s institution.

This article has been published as part of *BMC Systems Biology *Volume 7 Supplement 4, 2013: Selected articles from the IEEE International Conference on Bioinformatics and Biomedicine 2012: Systems Biology. The full contents of the supplement are available online at http://www.biomedcentral.com/bmcsystbiol/supplements/7/S4.
